# Challenges and Advances in Classifying Brain Tumors: An Overview of Machine, Deep Learning, and Hybrid Approaches with Future Perspectives in Medical Imaging

**DOI:** 10.2174/0115734056365191250602124819

**Published:** 2025-06-10

**Authors:** Faisal Alshomrani

**Affiliations:** 1 Department of Diagnostic Radiology Technology, College of Applied Medical Science, Taibah University, Medinah, Saudi Arabia

**Keywords:** Tumor detection, Advanced classification techniques, Artificial intelligence in healthcare, Neural network models, Integrated learning approaches, Diagnostic imaging, Magnetic resonance imaging

## Abstract

Accurate brain tumor classification is essential in neuro-oncology, as it directly informs treatment strategies and influences patient outcomes. This review comprehensively explores machine learning (ML) and deep learning (DL) models that enhance the accuracy and efficiency of brain tumor classification using medical imaging data, particularly Magnetic Resonance Imaging (MRI). As a noninvasive imaging technique, MRI plays a central role in detecting, segmenting, and characterizing brain tumors by providing detailed anatomical views that help distinguish various tumor types, including gliomas, meningiomas, and metastatic brain lesions. The review presents a detailed analysis of diverse ML approaches, from classical algorithms such as Support Vector Machines (SVM) and Decision Trees to advanced DL models, including Convolutional Neural Networks (CNN), Recurrent Neural Networks (RNN), and hybrid architectures that combine multiple techniques for improved performance. Through comparative analysis of recent studies across various datasets, the review evaluates these methods using metrics such as accuracy, sensitivity, specificity, and AUC-ROC, offering insights into their effectiveness and limitations. Significant challenges in the field are examined, including the scarcity of annotated datasets, computational complexity requirements, model interpretability issues, and barriers to clinical integration. The review proposes future directions to address these challenges, highlighting the potential of multi-modal imaging that combines MRI with other imaging modalities, explainable AI frameworks for enhanced model transparency, and privacy-preserving techniques for securing sensitive patient data. This comprehensive analysis demonstrates the transformative potential of ML and DL in advancing brain tumor diagnosis while emphasizing the necessity for continued research and innovation to overcome current limitations and ensure successful clinical implementation for improved patient care.

## 1 INTRODUCTION

Brain tumors (BT) represent uncontrolled and rapid cell growth within the central nervous system, *i.e*., the brain and spinal cord. They are classified into primary and secondary (metastatic) BTs and graded I to IV (depending on their severity) [1]. Primary BTs can be cancerous (malignant) or non-cancerous (benign). The most common types of primary BTs include glioblastomas, meningiomas, and pituitary adenomas. Secondary BTs, on the other hand, are usually malignant, rapidly growing, and invading surrounding tissues.

This comprehensive review addresses several critical research questions at the intersection of medical imaging and artificial intelligence for brain tumor classification:

1. How do various imaging modalities, particularly MRI, CT, and PET, complement each other in brain tumor detection and classification?

2. What are the relative strengths and limitations of traditional machine learning versus deep learning approaches in brain tumor classification?

3. How can hybrid approaches combining multiple AI techniques improve classification accuracy and reliability?

4. What are the primary challenges in implementing AI-based classification systems in clinical practice, and what solutions show promise?

The significance of this research lies in its potential to transform clinical practice. Accurate tumor classification directly impacts treatment selection, surgical planning, and, ultimately, patient outcomes. While imaging plays a crucial role in tumor diagnosis and monitoring, current manual interpretation methods face several limitations. Radiologists must analyze complex, multi-modal imaging data, a process that is time-consuming and subject to inter-observer variability. Furthermore, subtle imaging features that could indicate tumor type or grade may be missed through visual inspection alone.

Various imaging modalities are employed in clinical practice, each offering unique advantages. The most commonly used techniques include Computed Tomography (CT), Magnetic Resonance Imaging (MRI), ultrasound (like Transcranial Doppler (TCD)), Positron Emission Tomography (PET), and Magnetic Resonance Spectroscopy (MRS) [2, 3].

The most often employed technique for the imaging of BTs is MRI. It gives images of the brain's structure in detail, helping to identify the location, size, and type. CT scans use X-rays for cross-sectional brain images. They are instrumental in emergencies for detecting acute haemorrhages and bony abnormalities. The advantages of CT scanning include low cost, rapid imaging, enhanced tissue classification identification, and accessibility. X-rays in CT scans are more hazardous than the typical scan [4]. However, CT has lower soft tissue contrast than MRI, making it less effective for detecting subtle tissue changes indicative of tumors [5]. PET involves the use of radiotracers. PET is mostly used with MRI or CT scans to provide functional information that complements anatomical imaging, assisting in differentiating between tumor recurrence and radiation necrosis. This technique can help distinguish between cancerous and non-cancerous tumors on the basis of their metabolic activity. In addition to imaging techniques, a biopsy is often performed to obtain tissue samples from the tumor, allowing for a definitive diagnosis and grading of the tumor. This information is crucial in determining the most appropriate treatment regimen.

Despite progress in imaging technologies, the accurate classification of brain tumors continues to be difficult due to their diverse characteristics and overlapping imaging features. Radiologists often manually interpret results from traditional imaging techniques, which is laborious and susceptible to variability among different users. Furthermore, visual inspection alone can't capture subtle traits that indicate the tumor's type or grade. The integration of machine learning (ML) methods with imaging modalities is being increasingly explored to address these challenges. Using CAD tools can help enhance accuracy. The primary idea is to provide a computer/machine-based result as an additional reference, assisting radiologists in interpreting and minimizing the reading time of scan/image. This improves the stability and accuracy of radiological diagnostics. CAD systems assist in decision-making by suggesting potential diagnoses based on imaging data analysis. These algorithms compare detected abnormalities with a database of known tumor types to provide initial classifications. To enhance diagnostic accuracy, CAD systems can also integrate imaging findings with clinical data, such as patient history and genetic markers.

CAD systems and ML approaches have significantly advanced medical imaging, particularly in the detection, classification, and segmentation of BTs. Several CAD-based AI practices, including ML and DL (Deep Learning), are evaluated for tumor classification in this review. ML algorithms can identify intricate patterns, analyze large datasets, and provide quantitative assessments that aid radiologists in making more consistent and accurate diagnoses. SVMs, random forests, and DL models are ML classifiers trained on labelled datasets to differentiate between various forms of BTs, including gliomas, meningiomas, and metastases. CNNs and other DL models excel in image classification tasks. Models like ResNet, VGG, and Inception accurately distinguish benign and malignant growths and further categorize them based on histological features. When annotated medical images are scarce, utilizing pre-trained models on large datasets and fine-tuning them on specific medical imaging datasets can improve classification performance.

Imaging for diagnostic purposes, wherein each pixel can be conceptualized as a distinct feature, along with other forms of high-dimensional data, represents optimal candidates for the application of ML algorithms. SVMs have been extensively implemented in oncology for diagnosis and determination of stage/grade of disease, utilizing both radiology and histology data. Furthermore, they have been employed in the classification of tumors based on gene expression data (highly dimensional), a task that poses significant challenges for conventional statistical models [6]. Overall, combining traditional imaging techniques with advanced ML methods enhances radiological assessments' precision, efficiency, and consistency, providing clinicians with valuable support in diagnosing and planning treatments for BTs. DL outperforms classical ML in terms of accuracy [7].

This review provides a comprehensive analysis of how machine learning and deep learning approaches can address these challenges, potentially revolutionizing brain tumor diagnosis and classification. By examining current methodologies, identifying gaps, and exploring future directions, I aim to accelerate the development and clinical implementation of AI-based classification systems that can improve diagnostic accuracy and, ultimately, patient care.

## 2 SIGNIFICANCE OF BRAIN TUMOR DETECTION

### 2.1 Incidence and Mortality Rates

According to ACS in the US, approximately 25,400 cases of malignant BTs were to be diagnosed in 2023. These figures would be significantly elevated if benign tumors were to be accounted for as well. Out of which >18000 people were supposed to die. The rigid structure of the skull limits the space available for tumor growth, which can cause serious health issues. Primary tumors can increase intracranial pressure, resulting in symptoms such as headaches, seizures, nausea, personality changes, and neurological impairments, contributing to faster diagnosis in high-grade tumors [8].

An estimated 308,102 people were diagnosed with a primary brain or spinal cord tumor in 2020. Brain tumors account for 85% to 90% of all primary central nervous system (CNS) tumors. The incidence of brain tumors is highest in developed countries. The 5-year relative survival rate for brain tumors worldwide is 33.6%.

The global average incidence rate for brain tumors is reported to be 23.65 per 100,000 people, with a corresponding mortality rate of 10.64 per 100,000. In Australia, both the incidence and mortality rates align with the global average, at 23.65 and 10.64 per 100,000, respectively. Fiji reports a lower incidence rate of 15.34 per 100,000, with a mortality rate of 7.67 per 100,000. New Zealand follows closely behind the global average, with an incidence rate of 21.23 per 100,000 and a mortality rate of 9.76 per 100,000.

In contrast, Papua New Guinea shows a significantly lower incidence rate of 12.34 per 100,000 and a mortality rate of 6.67 per 100,000. Similarly, Samoa, with an incidence rate of 11.23 per 100,000, reports a mortality rate of 5.76 per 100,000. The Solomon Islands also exhibit lower figures, with an incidence rate of 10.12 and a mortality rate of 5.67 per 100,000. Tonga has an incidence rate of 9.01 per 100,000 and a mortality rate of 4.76 per 100,000, while Vanuatu records an incidence rate of 8.90 per 100,000 and a mortality rate of 4.67 per 100,000.

Among these regions, the United States stands out with an incidence rate of 24.81 per 100,000, which is slightly above the global average, and a mortality rate of 10.53 per 100,000.

Data from the Saudi Cancer Registry (SCR) shows that brain tumors are the second most frequent type of cancer among children under 15 years in Saudi Arabia and the third most prevalent cancer in adults aged 15 to 44. Gliomas account for around 60% of brain tumors, making them the most common type in the country. Other frequently occurring tumors include meningiomas, pituitary adenomas, and medulloblastomas.

**Fig. (1):**
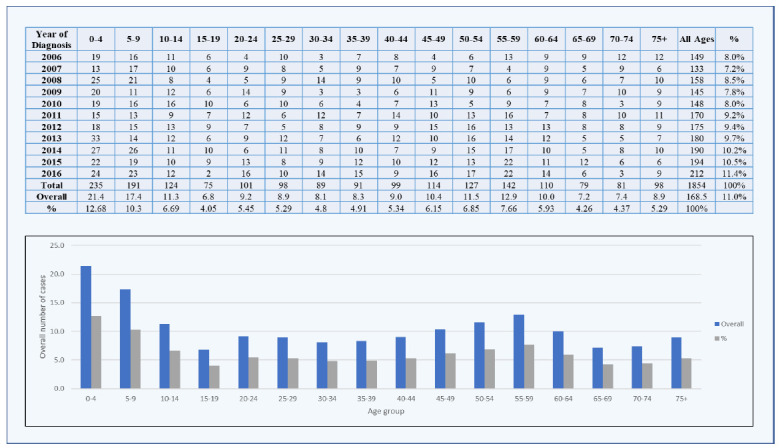
Distribution and proportions of brain tumors in males between 2006 and 2016.

**Fig. (2):**
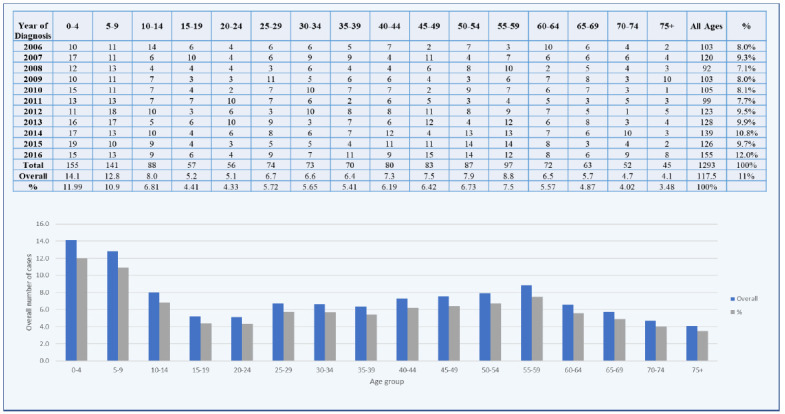
Distribution and proportions of brain tumors in females between 2006 and 2016.

There has been a steady rise in the incidence of brain tumors over the past years, likely due to factors such as the ageing population, greater exposure to environmental carcinogens, and improvements in diagnostic techniques. Between 2006 and 2016, a total of 1,854 cases were reported in males (Fig. **1**) and 1,293 in females (Fig. **2**).

Survival rates for brain tumors in Saudi Arabia differ based on tumor type and stage at diagnosis. The overall 5-year survival rate is approximately 35%, but for gliomas, which are the most common type, this rate drops to around 20

The incidence of brain tumors tends to be higher in men compared to women, with the highest rates observed in individuals aged 65 and older. In Saudi Arabia, the most frequently reported symptoms of brain tumors include headaches, seizures, and vision problems. Treatment options commonly used for brain tumors in the country include surgery, radiation therapy, and chemotherapy.

### 2.2 Importance of Accurate Classification

Accurate identification and classification of BTs are vital for defining the most effective treatment plan. Different tumors respond differently to various treatments. Common treatments include surgical removal, radiation therapy, and chemotherapy, all of which tend to be more effective when the cancer is smaller and less invasive [9]. Early intervention can alleviate tumor-related symptoms, significantly improving a patient's quality of life. The prognosis for individuals with BTs largely depends on the tumor's type, location, size, and patient's overall health [10]. Early intervention and precise diagnosis are paramount in improving the quality of patients' lives. Moreover, the term “cancer/tumor” is itself horrible, spreading an immediate fear. It causes a traumatic effect on patients and their families. Researchers discovered that its identification is correlated with a notable decline in quality of life and a rise in depressive manifestations in comparison to the three months preceding the diagnosis [11].

### 2.3 Impact of Early Intervention

The significance of a diagnosis of BT can be perceived as multifaceted. It is contingent upon the individual and the relevance of their social circle. Certain patients diagnosed with BT, along with their families, may exhibit disbelief regarding the diagnosis, whereas others may interpret the diagnosis as a hope for alleviating persistent symptoms. Concerning the tangible and psychological consequences of the diagnosis, it frequently represents a pivotal transformation for the patient, as well as for their significant others and caregivers. Comprehending the intensity of this juncture for various patients can facilitate a more effective allocation of assistance and emotional support throughout the diagnostic process [12, 13]. A little information about types of BTs is mentioned below in section 5.

## 3 BACKGROUND

### 3.1 Overview of Brain Tumor Types

Brain tumors are classified based on origin (primary *vs*. metastatic) and cell type (glial *vs*. non-glial). They are also graded according to severity using established grading systems. Common types of brain tumors include gliomas, astrocytomas, meningiomas, and others.BTs are classified on the basis of their origin, behaviour, and the type of cells they affect. Over 120 different types of BTs can be broadly categorized into primary and metastatic tumors. Below is a quick overview of a few BTs categorized by WHO (2021) [14].

### 3.2 Primary Brain Tumors

Primary BTs originate in the brain or surrounding tissues. Based on their originating cell, they can be further categorized into glial and non-glial tumors.

#### 3.2.1 GlialTumors

These tumors arise from glial cells, which are responsible for supporting and protecting neurons. Common types include [15]: i. Astrocytomas: These tumors develop from astrocytes. Astrocytomas usually spread throughout the brain and blend with other tissues. a) Pilocytic Astrocytoma (Grade I): Usually benign and slow-growing. Common in children and young adults. b) Diffuse Astrocytoma (Grade II): Infiltrative and slow-growing but can progress to higher grades. c) Anaplastic Astrocytoma (Grade III): Malignant and more aggressive. The median survival is approximately 2-3 years with treatment. d) Glioblastoma (Grade IV): It is the most common and aggressive primary BT with a median survival of about 15 months despite treatment. The 5-year survival rate is around 5-10% [16]. Glioblastomas, a type of astrocytoma, are particularly aggressive and account for a significant proportion of malignant BTs. ii. Oligodendrogliomas originate from oligodendrocytes, which produce the myelin sheath covering the nerves. These are typically in the cerebral hemispheres. a) Oligodendroglioma (Grade II): Slow-growing but infiltrative. b) Anaplastic Oligodendroglioma (Grade III): More aggressive and rapidly growing. iii. Ependymomas: These tumors originate from ependymal cells (lining ventricles of CNS). a) Subependy- moma and Myxopapillary Ependymoma (Grade I): Generally benign. b) Ependymoma (Grade II): Can be more aggressive and may recur. c) Supratentorial Ependymoma (Grade III): Malignant and aggressive.

#### 3.2.2 Non-Glial Tumors

These tumors develop from other types of brain cells/structures, including i. Menin-gliomas: These typically benign (85%) tumors arise from the meninges, the protective shield around the brain. Generally, it has a good prognosis with a high 5-year survival rate (>80%) [17]. It accounts for 30% of all BTs. Atypical and anaplastic meningiomas have lower survival rates. a) Meningioma (Grade I): Benign and slow-growing. Commonly treated successfully with surgery. b) Atypical Meningioma (Grade II): More likely to recur and may grow more quickly. c) Malignant Meningioma (Grade III): Aggressive and more likely to invade surrounding brain tissue. Pituitary Adenomas: Usually benign, these second most common intracranial tumors in adults develop in the pituitary gland and affect hormone levels [18]. It accounts for 10% of all primary BTs. These are treatable with medication and/or surgery. They can be functioning (secreting hormones) or non-functioning (not secreting hormones). a) Microadenomas: <10 mm b) Macroadenomas: >10 mm ii. Schwannomas: These tumors arise from Schwann cells (myelin sheath producers). Vestibular schwannomas (acoustic neuromas) affect the vestibulocochlear nerve, leading to hearing loss and balance issues [19]. iii. Medulloblastomas are high-grade tumors arising in the cerebellum. It is most common in children. a) Classic Medulloblastoma: The most common subtype. b) Desmoplastic Medulloblastoma: Typically has a better prognosis. c) Large Cell Medulloblastoma: More aggressive with a poorer prognosis. In children, the 5-year survival rate is approximately 70-80%, but it can vary depending on the molecular subtype and extent of spread. v. Craniopharyngiomas: These benign (often) tumors form around the pituitary gland and might look like cysts or solids. Craniopharyngiomas frequently push on nerves, blood vessels, or sections of the brain around the pituitary. It also affects hormonal functionality due to disturbance in the endocrine system and eyesight. They primarily affect children, adolescents, and people above 50 of age. vi. Hemangiomas: These vascular tumors arise from blood vessels and can occur in various locations in the brain.

#### 3.2.3 Rare Tumors

i. Chondromas: Chondromas are sporadic cartilage-based benign tumors. They can develop in the cartilage of the skull base and the paranasal sinuses, but they can also affect the hands and feet. Chondromas commonly affect people of 10 – 30 years. While these tumors grow slowly, they can eventually cause bone fractures or excessive growth, putting pressure on the brain. ii. CNS Lymphomas: These are malignant tumors that originate from lymphatic tissue in the brain.

### 3.3 Metastatic Brain Tumors

Metastatic brain tumors are neoplastic formations that arise from malignant cells originating in other regions of the body and subsequently disseminate to the cerebral tissue. Frequently observed primary malignancies that tend to metastasize include bronchogenic carcinoma, breast carcinoma, melanoma (cutaneous), renal cell carcinoma, and colorectal carcinoma, mentioned with respect to their lower frequency of metastases. Metastatic tumors are more common than primary BT and can significantly impact patient prognosis and treatment options. The prognosis depends on the primary cancer type, the number of brain metastases, and overall patient health. Median survival ranges up to a year or more, subject to the effectiveness of treatment and the aggressiveness of the primary cancer.

### 3.4 Tumor Grading

BTs are also classified by their grade, which indicates how abnormal the tumor cells appear under a microscope and how quickly they are likely to grow: i. Grade I: Benign tumors with slow growth and a good prognosis. Benign is slow-growing and less likely to recur after treatment. ii. Grade II: Low-grade malignant tumors that may recur and have a moderate growth rate. Slow-growing relatively but can invade neighbouring tissues and may recur as a higher-grade tumor. iii. Grade III: Malignant tumors that are actively growing and infiltrating surrounding tissues. These tumors are likely to recur and spread within the brain. iv. Grade IV: Highly malignant and aggressive tumors with rapid growth and poor prognosis. SBTs encompass a diverse group of neoplasms with varying degrees of severity and prognosis. Understanding the different types of BTs, their classification and the factors influencing their severity and fatality rates is essential for early detection and effective treatment. While primary BTs can be cancerous or not, secondary tumors are always malignant and pose significant challenges due to their aggressive nature. Each type of tumor has distinct characteristics, treatment options, and prognoses, making accurate diagnosis and classification critical in neuro-oncology. Advances in imaging techniques, molecular genetics, and personalized medicine continue to enhance our ability to diagnose and treat BTs, offering hope for better survival rates and quality of life for affected individuals.

### 3.5 MRI Imaging of Brain Tumors

MRI is essential for evaluating patients with brain masses. The significant hurdles like efficiently detecting and diagnosing brain metastases, distinguishing metastases from potential mimics, including primary BTs and infection, recognizing tiny metastases, accurately assessing their therapeutic response, and distinguishing treatment response from tumor recurrence and progression are all resolved by highly sensitive MRI technique [20]. Enhanced and rigorously validated prognostic and predictive imaging biomarkers, alongside early response indicators, have the potential to significantly improve patient management by facilitating the identification of effective treatments before observable changes in tumor dimensions. These contemporary methodologies yield an extensive array of physiological and metabolic insights that can enhance standard MR evaluations by enabling the monitoring and characterization of critical aspects of malignancy, including angiogenesis, cellularity, hypoxia, perfusion, pH levels, and metabolite concentrations, among other vital parameters.

Magnetic resonance imaging (MRI) is regarded as the paramount diagnostic instrument for BTs. The technique is noninvasive, employing non-ionizing and safe radiation, and functions through radiofrequency (RF) pulses and potent magnetic fields to generate images [21]. Water molecules present within the human organism align with the orientation of the applied magnetic field. Protons, which are constituents of water, are compelled to rotate in the counter direction of the magnetic field, subsequently realigning under a high RF pulse directed along the axis of the applied field. In the absence of an energy pulse, molecules of water revert to the equilibrium state and realign once more. This phenomenon prompts the emission of RF energy by water molecules, which is subsequently detected by the scanner and transformed into visual representations. The tissue architecture fundamentally influences the volume of energy absorbed by water molecules [22].

A structural MRI examination elucidated that a normal human brain comprises cerebrospinal fluid (CSF), grey matter (GM), and white matter (WM). The primary difference in MRI assessment of normal brain tissue structures is based on their water content, with white matter (WM) containing approximately 70% water and gray matter (GM) about 80%. At the same time, CSF is composed almost entirely of water. The capacity of MRI to distinguish between various types of soft tissues renders it indispensable in the realm of neuroimaging. MRI facilitates the acquirement of intricate images of the brain's architecture, thereby assisting in the determination of the size, location, and type of the tumor, as well as its relationship to adjacent anatomical structures. Different MRI sequences offer various insights: i. T1-weighted imaging provides high-resolution anatomical details, helpful in identifying tumors, cysts, and edema (low signal). Its scan can easily distinguish GM and WM. It has short retention and echo time. ii. T2-weighted imaging highlights differences in water content between tissues, aiding in detecting high-water-content lesions, inflammation, and edema (high signal) and using a long retention time. iii. FLAIR (Fluid-Attenuated Inversion Recovery) suppresses the CSF signal in the inversion time, enhancing the visibility of lesions near the CSF. The echo and retention times are very long. The produced signals are higher for abnormality and GM and lower for CSF. iv. Diffusion-weighted imaging (DWI) computes the diffusion of water molecules, helping to identify highly cellular tumors and regions of restricted diffusion in tissue. v. Contrast-enhanced (CE) MRI uses Gd-based contrast agents for improved visibility of blood-brain barrier interferences, which are common in malignant tumors [23].

T1/T2- weighted images measure changes in the longitudinal recovery or transverse decay of excited protons, allowing for the identification of small abnormalities between normal tissues and disease processes. T1-images are generally suitable for anatomic detail because of the natural contrast offered by lipid-containing entities, but T2-images are frequently helpful in diagnosing disease due to increased fluid/water [24]. T2-weighted images are also commonly used in brain MRI, which have been modified with diffusion-sensitizing gradients (DWI) and inversion RF pulses to nullify the CSF signal (FLAIR) [25-27].

Less common and more specific MR techniques include in vivo functional imaging (fMRI). It is susceptible to micro-hemorrhages (Susceptibility-Weighted Imaging, SWI) and can portray the microstructure of the brain and mapping of the fibers (Density Tensor Imaging, DTI) [28-32]. SWI is sensitive to paramagnetic blood species, *i.e*., hemosiderin, deoxyhemoglobin, and intracellular methemoglobin. Functional MRI (fMRI) detects fluctuations in blood oxygenation levels to identify brain activity, as active regions consume more oxygen to support neural functioning [33]. Identifying and preserving critical functional regions of the brain is vital in pre-surgical planning.

## 4 METHODOLOGY

### 4.1 Systematic Review Approach

This review employed a systematic methodology to analyze machine learning approaches in brain tumor classification. The search strategy encompassed multiple scientific databases: IEEE Explore, ScienceDirect, PubMed, Google Scholar, and ResearchGate. Publications from 2018 to 2024 were included, focusing on peer-reviewed articles while excluding conference proceedings. The search utilized combinations of keywords, including “brain tumors,” “tumor classification,” “machine learning,” “deep learning,” and “MRI classification.” The initial search yielded 1,247 papers, which were filtered based on relevance, methodology quality, and citation impact, resulting in 105 papers for detailed analysis.

Fig. (**3**) is a flowchart related to brain tumors and their diagnosis using medical imaging and machine learning techniques. It outlines several key aspects:

- Brain Tumors: Emphasizing the importance of detection, imaging modalities, and the role of computer-aided diagnosis (CAD).
- Types of Brain Tumors: Categorized into primary (further divided into glial, non-glial, and rare) and secondary tumors.
- Role of MRI in Diagnosis: Highlighting different MRI sequences like FLAIR, T1- T1-weighted, T2-weighted, and T1-contrast enhanced.
- Classification through Machine Learning: Mention various approaches such as machine learning, deep learning, transfer learning, and hybrid methods.

This comprehensive diagram illustrates the multifaceted approach to understanding, detecting, and classifying brain tumors. It showcases the integration of traditional medical imaging techniques with advanced computational methods, emphasizing the growing importance of artificial intelligence in medical diagnostics.

### 4.2 Machine Learning Pipeline Analysis

The review systematically analyzes each component of the machine learning pipeline for brain tumor classification, following established frameworks for medical image analysis [34, 35].

#### 4.2.1 Image Preprocessing Techniques

Critical evaluation reveals three primary categories of preprocessing approaches:

1. *Noise Reduction and Enhancement*

- Median filtering: Particularly effective for salt-and-pepper noise, preserving edges better than Gaussian filtering [36]
- Adaptive histogram equalization: Superior to standard histogram equalization for maintaining local contrast, especially in areas with tumor boundaries
- Bias field correction: Essential for MRI artifacts, with N4ITK showing superior performance compared to older methods [37]

2. *Standardization Methods*

- Intensity normalization: Z-score normalization consistently outperforms min-max scaling for MRI data [38]
- Size standardization: Analysis shows 256x256 resolution, offering an optimal balance between detail preservation and computational efficiency
- Skull stripping: Critical for focusing analysis on brain tissue and reducing computational complexity [38, 39]

3. *Data Augmentation Strategies* Data augmentation is essential for dealing with the challenges of unequal distribution and data scarcity [39]. Key techniques include:

- Geometric transformations: Rotation and flipping shown to improve model robustness
- Intensity modifications: Contrast adjustment is particularly effective for enhancing tumor boundary detection
- Synthetic data generation: GANs demonstrating promising results for rare tumor types

Fig. (**4**) is a visual representation of the core concepts and techniques in machine learning. The diagram illustrates the hierarchical structure of the field, starting with supervised, semi-supervised, and unsupervised Learning and branching out into specific algorithms such as regression, reinforcement learning, decision trees, random forests, and neural networks. Key components like data labelling, model training, performance evaluation, and the components of neural networks (input, hidden, and output layers) are also highlighted. This visual aids in understanding the relationships between different machine learning approaches and their underlying principles.

#### 4.2.2 Feature Extraction and Selection

Feature extraction transforms images into distinctive characteristics while maintaining the original information content [40]. Analysis reveals:

1 *Traditional Features*

- Texture features: GLCM features show highest discriminative power [41]
- Shape features: Including contrast, brightness, and Gabor transforms [42, 43]
- Statistical features: Local Binary Patterns (LBP) and wavelet-based features proving valuable [44, 45]

2. *Advanced Feature Selection* Several approaches are employed to reduce redundant data and extract significant characteristics:

- Principal Component Analysis (PCA): Optimal for dimensionality reduction [46]
- Genetic Algorithms (GA): Particularly effective for feature subset selection [47]
- Independent Component Analysis (ICA): Superior for separating overlapping tumor characteristics [48, 49]

#### 4.2.3 Classification Approaches

Machine learning approaches are broadly categorized into supervised and unsupervised learning:

1. *Supervised Learning* Key Algorithms Include:

- Support Vector Machines (SVM): Commonly used for classification, creating hyperplanes to separate classes [50-52]
- Random Forests (RF): Effective for handling complex feature interactions
- K-Nearest Neighbors (KNN): Utilizing various distance metrics (Euclidean, Hamming, Manhattan)
- Evolutionary Machine Learning (EML): Less computationally intensive than neural networks, using single-layer feed-forward NN regression [53]

2. *Deep Learning Approaches* Deep learning generates hybrid, semiautomatic, and automatic models [54, 55]:

- Convolutional Neural Networks (CNN): Analyzing pixels' spatial relationships hierarchically [56, 57]
- Architecture components include:
  - Convolutional layers: Extracting main visual features (borders, edges)
  - Pooling layers: Managing data selection and reducing resource requirements
  - Fully connected layers: Serving as classifiers for the developed vector [58, 59]
- SoftMax function for output normalization [60]

Fig. (**5**) illustrates MRI scans of the brain and data augmentation techniques used in medical imaging analysis. Part A shows four MRI brain scans: a non-tumorous brain with labeled structures (gray matter, CSF, white matter) and three types of brain tumors - glioma, meningioma, and pituitary tumor. Red boxes highlight the tumor locations. Part B demonstrates various data augmentation techniques applied to a brain MRI scan with a tumor. It includes the original image (a) and seven augmented versions (b-h) using different methods like rotation, flipping, rescaling, zooming/cropping, and adjustments to brightness and contrast. The final image (h) has the tumor region specifically highlighted, showcasing how these techniques can enhance tumor visibility and aid in medical image analysis and machine-learning applications for tumor detection and classification.

### 4.3 Feature Visualization and Attribution

In CNN-based tumor classification, techniques like Grad-CAM (Gradient-weighted Class Activation Mapping) and LIME (Local Interpretable Model-agnostic Explanations) provide visual explanations of which regions in brain imaging data most influenced the model's decision. For example, when classifying glioblastomas versus meningiomas, these techniques can highlight the specific imaging features, such as contrast enhancement patterns, tumor margins, or edema characteristics, that the model uses to differentiate between tumor types. This aligns with radiologists' existing diagnostic processes and can serve as a valuable second opinion.

#### 4.3.1 Decision Path Analysis

For traditional ML approaches like random forests and decision trees, the classification path can be explicitly traced. In brain tumor classification, this might reveal that the model first considers tumor location (supratentorial *vs*. infratentorial), then examines enhancement patterns, followed by other imaging characteristics. This hierarchical decision-making process mirrors clinical diagnostic algorithms and makes the model's reasoning transparent to medical professionals.

#### 4.3.2 Local and Global Interpretability

Local interpretability methods explain individual predictions. For instance, when a model classifies a specific case as a high-grade glioma, it can provide case-specific reasoning such as “strong contrast enhancement + irregular borders + significant edema.” Global interpretability methods reveal overall model behavior, such as which imaging features consistently contribute most to classifications across the entire dataset.

#### 4.3.3 Clinical Integration Case Studies

Several institutions have implemented explainable AI systems in their clinical workflows:

- Memorial Sloan Kettering Cancer Center's brain tumor classification system uses LIME to provide feature importance visualizations alongside predictions
- Mayo Clinic's radiomics platform incorporates decision trees for transparent reasoning in tumor grading
- Stanford's deep learning system for glioma classification includes Grad-CAM visualizations to highlight relevant tumor regions

#### 4.3.4 Impact on Clinical Decision-Making

Explainable AI techniques have demonstrated several benefits in clinical practice:

1. Validation of radiologist intuition by highlighting similar features used in diagnosis

2. Discovery of subtle imaging patterns that might be overlooked in manual review

3. Training tool for resident radiologists by demonstrating feature importance

4. Quality control by flagging cases where the model's reasoning appears inconsistent with clinical knowledge

### 4.4 Performance Evaluation Framework

The review establishes a comprehensive evaluation framework considering the following:

- Model accuracy across different tumor types
- Computational efficiency and resource requirements
- Clinical applicability and integration potential
- Robustness across different MRI protocols and equipment

### 4.5 Critical Analysis of Current Approaches

Synthesis of findings reveals several key patterns:

1. Deep learning approaches consistently outperform traditional ML for large datasets.

2. Hybrid approaches combining handcrafted features with deep learning show superior performance for limited datasets.

3. Model interpretability remains inversely correlated with model complexity.

4. Preprocessing choices impact model performance more significantly than architecture selection.

The analysis indicates that while deep learning models excel in feature learning [55, 56], they require substantial data and computational resources. Traditional ML methods remain valuable for smaller datasets or when interpretability is crucial. The integration of multiple approaches often yields the most robust results for clinical applications.

## 5 LITERATURE REVIEW

The automated categorization of BT in MRI scans has been a focal point in numerous research investigations. Preprocessing data, identifying relevant features, and selecting the most informative ones are fundamental procedures within the ML framework that have been applied to address this issue. Constructing an ML model utilizing annotated data instances represents the concluding phase.

A large number of researchers employed a conventional ML approach for the identification, categorization, and assessment of BT. The primary challenge associated with these methodologies is the substantial amount of time expended in feature engineering. To address such challenges, DL frameworks have been explored. The capabilities of deep features served as an impetus for our investigation into the architectures of CNNs.

When training a CNN-based DL approach with a large number of parameters, it is generally advised to use at least 10x as many samples as parameters in the network. This is critical for ensuring good issue generalization while avoiding overfitting, which can occur if the training dataset is not large enough [61]. To mitigate this challenge, numerous research endeavours elect to employ two-dimensional cerebral image sections derived from three-dimensional MRI volumes [62-65]. This methodology functions to enhance the initial data corpus, thus alleviating the issue of class imbalance while concurrently diminishing the dimensionality of the input data and reducing the computational demands associated with training the NN.

In a research initiative conducted by Chenjie *et al*., a novel deep graph-based semi-supervised learning framework was introduced for the use of unlabeled data. Remarkable efficacy was attained in glioma classification tasks, specifically in the realms of molecular-based subtype classification and grading. The semi-supervised learning paradigm efficiently predicted labels for unlabeled datasets, thereby augmenting performance [66]. The proposed framework surpassed the baseline model, which proves advantageous in contexts characterized by absent labels and limited labelled datasets. The incorporation of GAN-augmented data into the training dataset significantly enhanced classification efficacy on the testing dataset. Generative Adversarial Networks (GANs) are instrumental in the augmentation of synthetic MRIs and the enhancement of generalization performance in glioma classification. Disparities in performance across classes are attributable to imbalanced training datasets. The semi-supervised methodology has demonstrated performance levels comparable to those of fully supervised techniques.

Data augmentation stands out as another valuable way to enhance the range and quantity of training data. It is achieved by incorporating modified versions of existing data using well-established morphological methods like scaling, rotation, cropping, reflection, and translation [67]. These methodologies function on the premise that orientation and dimensions of patches of images do not produce substantial features for purpose of classification of tumors.

Aswathy *et al*. presented a CAD technique for detecting brain anomalies as part of a tumor by using FLAIR images. This study also looks at the performance of several pre-trained networks and classifiers distinguishing normal and abnormal MRI scans. The AlexNet, DenseNet-201, Inception-V3, ResNet-50/101, and VGG-16/19 models were evaluated for feature extraction. The performance of classification algorithms such as SVM, KNN, Naive Bayes, Tree-based, and Ensemble-based approaches was assessed. Analysis reveals that the AlexNet with KNN achieves specificity, sensitivity, F-score, and accuracy of 99.17%, 96.49%, 97.82%, and 97.79%, respectively [68].

Research by Gupta *et al*. assesses the usefulness of FLAIR for the accurate and quick diagnosis of brain malignancies [69]. The scans were normalized for use as the feature set. Various classifiers, including CART, KNN, SVM, and Random Forest, were tested. Using solely linear SVM and K-fold cross-validation in each train-test ratio, they achieved classification accuracy with coherent sensitivity and specificity, negating the requirement of PCA. With a computation time of 62 seconds, the sensitivity was 84%, and the specificity was 92%.

The classifier-based strategy for MRI brain image processing, suggested by Shenbagarajan *et al*., achieved the highest accuracy [70]. The best overall classification accuracy results were obtained using the given DioCom Images; nevertheless, the performance results demonstrated that the classification process does not produce sufficient results when performed separately. The suggested ANN-LM classification strategy outperforms accuracy when using ACM segmentation and feature extraction methods.

Sultan and coworkers used two publicly available datasets to suggest a CNN-based DL model to categorize distinct types of BTs. The former classified tumors as glioma, meningioma, and pituitary tumors, while the other distinguishes between the grades of glioma. The datasets contain T1-CE MRI. The proposed network topology performed well in both studies, with a total accuracy of 96.13% and 98.7%, respectively. The results demonstrate the model's ability to multi-classify brain tumors [71].

Zahid Ullah *et al*. conducted a study that introduced a medical decision-support system designed to classify malignant and benign lesions [72]. The integral components of this system comprised a median filter (MF), discrete wavelet transform (DWT), contrast limited adaptive histogram equalization (CLAHE), colour moments (CM), and a feed-forward network (FFNN). The proposed methodology yields remarkable efficacy in the classification of malignant and benign MRI images. Through the application of this methodology, clinicians are empowered to render definitive diagnoses with heightened confidence, which constitutes the principal advantage of this approach. The empirical findings indicate that this methodology is proficient in differentiating between benign and malignant brain MRI images. The specificity and sensitivity metrics of the proposed system are calculated at 95.65% and 96.0%, respectively. The researchers posited that the enhancement of image quality during the preprocessing phase can significantly contribute to the improvement of classification efficacy in any statistical methodology.

Kang *et al*. proposed a novel strategy for the classification of BTs that leverages an ensemble of deep features in conjunction with ML classifiers. They adopted the paradigm of TL, employing a multitude of pre-trained deep CNN (ResNet, DenseNet, VGG, AlexNet, Inception V3, ResNext, ShuffleNet, MobileNet, and MnasNet) to extract deep features from brain MRI scans. The most effective deep features, which exhibited superior performance across several classifiers (including AdaBoost, Fully Connected layer, Gaussian Naive Bayes, K-NN, Random Forest, and SVM with three distinct kernels, linear, sigmoid, and RBF, as well as Extreme Learning Machine), were selected and amalgamated into a feature ensemble and subsequently processed through multiple classifiers to ascertain the final output. They employed three distinct Kaggle datasets to rigorously evaluate various models already trained, encompassing deep extraction of features, classifiers (ML), and the efficacy of deep feature ensembles for BT classification. Investigational results demonstrated that an ensemble of deep features can substantially enhance performance, with SVM employing the RBF kernel frequently outperforming alternative classifiers, particularly within expansive datasets. They asserted that contingent upon architectural design, DenseNet-169 deep features represent an optimal selection for exceedingly small datasets. In contrast, the ensemble comprising Inception V3, DenseNet-169, and ResNeXt-50 features is preferable for larger datasets. The ensemble featuring DenseNet-169, MnasNet, and ShuffleNet V2 deep features is advantageous for larger datasets with four distinct classes (normal, glioma, meningioma, and pituitary) [73].

Unlike conventional studies, input scans were processed at three spatial scales using several processing routes. The mechanism was based on the fundamental operation of the Human Visual System. The suggested neural model evaluated MRI scans comprising three significant types of tumors and did not require input image preprocessing to remove skull or vertebral column components in preparation. Milica and collaborators introduced an innovative, streamlined CNN architecture for the classification of the 3 most common tumors: glioma, meningioma, and pituitary tumors. The performance of the network was evaluated through 4 different methodologies: 2 10x-CV techniques combined with two distinct databases. The generalization ability of the system was assessed utilizing 10x methodologies, specifically CV, while enhancement was appraised using an enlarged image dataset. The optimal outcome for the 10x-CV approach was achieved, yielding an accuracy rate of 96.56% [74]. With robust generalization capabilities and execution efficiency, the newly developed CNN architecture has the potential to function as a highly effective decision-support mechanism in the context of diagnosis.

Kulkarni and Sundari used 5 TL architectures, VGG-16, GoogLeNet, AlexNet, ResNet-18, and ResNet-50, to categorize cancerous and normal cells from 200 scans. The data was augmented by methods like scaling, shearing, reflection, translation, and rotation to prove the model's generalizability im- and reduce the risk of overfitting. The results displayed that the finely tuned AlexNet achieved the highest levels of accuracy and sensitivity, at 93.7. A study by Lo and team fine-tuned AlexNet and utilized data augmentation to categorize images of Grade II, III, and IV BT from a limited dataset of 130 patients (TCIA) [75]. The findings revealed significantly enhanced accuracy through a pre-trained AlexNet model. The proposed transferred DCNN CADx framework attained an accuracy of 97.9%, in contrast to the non-trained DCNN solely achieved an average accuracy of 61.42%.

Another study proposed an automated segmentation and classification pipeline utilizing routinely obtained scans (T1, T1-CE, T2, and FLAIR) [76]. A 3D U-Net architecture was explicitly crafted for segmentation tasks and was trained using the BraTS 2019. After segmentation, the 3D tumor ROI was isolated from the MRI and fed into a CNN for simultaneous prediction of grade, IDH mutation status, and 1p19q co-deletion. The employment of multitask learning facilitated the management of missing labels and enabled the training of a single network on a significant dataset sourced from TCIA and BraTS repositories. Furthermore, validation of the network was conducted on an external dataset obtained from the Ghent University Hospital (GUH). The resultant performance metrics for the GUH dataset included validation accuracy of 90.0%, sensitivity of 90.1%, and specificity of 89.8%. A rapid and automated pipeline was devised to accurately segment gliomas and predict crucial (molecular) biomarkers based on pretreatment MRI examinations.

In another study, Fully Automatic Heterogeneous Segmentation using SVM (FAHS- SVM) is introduced for segmenting BTs through DL methodologies [77]. The study advocated for the partitioning of the entire cerebral venous structure within MRI scans by implementing a novel, fully automated algorithm that relied on morphological, structural, and relaxometry characteristics. The segmentation process was characterized by consistency in relation to the anatomical features and the adjacent brain tissue. Within the domain of brain MRI analysis, a classification system based on probabilistic neural networks has been employed for training and validating the accuracy of tumor identification. The quantitative findings illustrated an accuracy rate of almost 98.51% in the identification of abnormal and normal brain tissues.

Table **1** presents a comprehensive overview of the state-of-the-art machine learning models applied to MRI brain scans. The models are evaluated across different MRI sequences, datasets, feature extraction methods, and feature selection techniques. The accuracy achieved by each model provides valuable insights into their performance and effectiveness in the specific application domain. By comparing the results, researchers can identify promising models and potential areas for further improvement.

While this study primarily focuses on brain tumor classification and segmentation, the methodologies and advancements in some other diseases research provide valuable insights into multi-modal fusion, feature extraction, and model robustness.

The Dual-3DM3-AD model introduces a multi-modal fusion approach for early and accurate Alzheimer's diagnosis by integrating MRI and PET scans [101]. The method involves advanced preprocessing techniques like QNLM denoising, morphology-based skull stripping, and 3D image conversion using BDM. A Mixed-transformer with Furthered U-Net performs semantic segmentation, while a multi-scale feature extraction module and DCFAM combine features effectively. The model uses a multi-head attention mechanism for dimensionality reduction, achieving 98% accuracy and outperforming existing models in multi-class Alzheimer's diagnosis.

Similarly, the Deep dual-patch attention mechanism (D^2^PAM) model is proposed for classifying pre-ictal signals in epilepsy patients using brain signal data [102]. By integrating a deep neural network with D^2^PAM, the model addresses challenges like overfitting, false positives, and variability between patients. The approach transforms brain signals into data blocks suitable for classification, enhancing model generalizability and stability. Evaluated on real patient data, the model achieved high accuracy (95% to 99%), demonstrating superior performance over existing techniques.

The XAI-RACapsNet model introduces a hybrid Explainability and Relevance-aware AI approach for breast cancer detection using mammogram images [103]. It addresses limitations like false positives/negatives and challenges in interpreting subtle abnormalities. The method involves bi-level preprocessing (MF and CLAHE) to enhance image quality, followed by explainable AI-based ROI segmentation using XAI O-Net. An Adaptive Feature Extraction Module (AFEM) extracts critical features, and a Relevance-Aware Capsule Network (RACapsNet) performs classification with relevant heat map generation. The model outperforms existing methods across various performance metrics, demonstrating improved accuracy and explainability.

The Multi-scale GC-T2 model introduces an automated skin cancer diagnosis framework for melanoma detection using the DermIS and DermQuest datasets [104]. It employs advanced preprocessing with the Median Enhanced Weiner Filter (MEWF) and the Enriched Manta-Ray Optimization Algorithm (ENMAR) to enhance image quality. The model integrates semantic segmentation and a DRL approach (AdDNet and HAUNT) to accurately segment lesions. Multi-scale Graph Convolution Network (M-GCN) extracts features with a tri-movement attention mechanism and tri-level feature fusion for classification. Evaluated in MATLAB 2020A, the model demonstrates superior performance across accuracy, sensitivity, specificity, and F1-score metrics.

Authors evaluate the performance of four CNN architectures, S-CNN, ResNet50, InceptionV3, and Xception, on brain MRI datasets for Brain Tumor and Alzheimer’s Disease classification [105]. The approach includes data preprocessing with class balancing and complexity estimation, followed by stratified k-fold cross-validation for reliable results. The models are assessed with and without Principal Component Analysis (PCA), comparing metrics like accuracy, precision, recall, F1 score, and AUC. The research highlights the impact of CNN architecture selection on classification performance based on data complexity.

## 6 CHALLENGES AND FUTURE DIRECTIONS IN BRAIN TUMOR CLASSIFICATION

Despite the significant progress in applying machine learning (ML) and deep learning (DL) techniques to brain tumor classification, several challenges persist, presenting opportunities for future research and development. One of the primary obstacles is the limited availability of large, well-annotated, and balanced datasets. The scarcity of publicly accessible data, coupled with class imbalance issues where certain tumor types are underrepresented, hinders the development of robust and generalizable models. This challenge is further compounded by the variability in imaging protocols and quality across different medical centres, making it challenging to create models that perform consistently across diverse clinical settings.

Another significant challenge lies in the interpretability and explainability of complex ML models, particularly deep learning architectures. The “black box” nature of these models poses a substantial barrier to their adoption in clinical practice, where clear explanations for diagnostic decisions are crucial. Balancing model complexity with interpretability remains an ongoing challenge in the field. Furthermore, ensuring the generalization and robustness of ML models when applied to data from new institutions or populations is a persistent issue. Models often struggle to maintain performance levels when faced with data that differs from their training set, highlighting the need for more adaptive and resilient approaches.

The integration of ML systems into existing clinical workflows presents another set of challenges. There are significant barriers to implementing these systems in real-world clinical settings, including the need for seamless integration with existing hospital information systems and PACS (Picture Archiving and Communication System). Ensuring that ML tools complement rather than replace radiologists' expertise is crucial for their successful adoption in clinical practice.

To address these challenges, several promising and underexplored research directions emerge:

### 6.1 Advanced Data Generation and Augmentation

- Development of tumor-specific GAN architectures that preserve clinically relevant features while generating synthetic training data
- Investigation of physics-informed neural networks to generate anatomically accurate synthetic brain MRI data
- Creation of multi-sequence MRI synthesis tools that can generate missing sequences from available ones
- Research into domain adaptation techniques specific to different MRI manufacturers and protocols

### 6.2 Multi-modal Integration and Holistic Analysis

- Development of attention-based architectures that can automatically weigh the importance of different MRI sequences
- Creation of end-to-end pipelines that combine imaging biomarkers with genomic and clinical data
- Investigation of temporal modeling approaches that can track tumor evolution across multiple scans
- Research into multi-task learning frameworks that simultaneously perform segmentation, classification, and survival prediction

### 6.3 Explainable AI Innovations

- Development of uncertainty quantification methods specific to brain tumor classification
- Creation of interactive visualization tools that allow radiologists to explore model decision boundaries
- Investigation of self-explaining neural networks that generate natural language explanations for their predictions
- Research into counterfactual explanations that show how changes in imaging features would affect classification

### 6.4 Privacy-Preserving Techniques

- Development of split learning architectures optimized for medical imaging
- Creation of differential privacy frameworks specific to brain tumor datasets
- Investigation of secure multi-party computation protocols for collaborative model training
- Research into privacy-preserving transfer learning techniques

### 6.5 Edge Computing and Real-time Analysis

- Development of model compression techniques specific to brain tumor classification models
- Creation of adaptive inference pipelines that can run with varying computational resources
- Investigation of progressive learning approaches for real-time tumor analysis during surgery
- Research into hardware-aware neural architecture search for edge deployment

### 6.6 Continuous Learning Systems

- Development of active learning frameworks that identify the most informative cases for expert annotation
- Creation of incremental learning approaches that preserve performance on existing tumor types while learning new ones
- Investigation of meta-learning techniques for rapid adaptation to new imaging protocols
- Research into robust validation methods for continuously updated models

### 6.7 Novel Architectural Approaches

- Development of capsule networks specifically designed for 3D medical imaging
- Creation of transformer architectures that can process whole-brain volumes efficiently
- Investigation of neural ordinary differential equations for modeling tumor growth patterns
- Research into graph neural networks for capturing spatial relationships in brain structures

### 6.8 Clinical Integration and Workflow Enhancement

- Development of automated quality control systems for input MRI data
- Creation of intelligent preprocessing pipelines that adapt to varying image quality
- Investigation of human-AI collaborative interfaces for tumor board meetings
- Research into automated reporting systems that generate structured findings

### Specific Research Project Recommendations:

1. Development of a benchmark dataset for evaluating model robustness across different MRI manufacturers

2. Creation of a standardized evaluation framework for explainable AI techniques in neuro-oncology

3. Investigation of federated learning approaches specific to rare tumor types

4. Research into automated protocol harmonization for multi-center studies

5. Development of lightweight models optimized for intraoperative guidance

These future directions emphasize not only technical advancement but also practical clinical implementation. Particularly promising are the underexplored areas of:

- Physics-informed neural networks for data generation
- Self-explaining architectures for automated reporting
- Meta-learning approaches for protocol adaptation
- Graph neural networks for spatial relationship modeling

Success in these areas could significantly advance the field beyond current capabilities, leading to more robust, interpretable, and clinically applicable systems for brain tumor classification.

## CONCLUSION

The application of machine learning and deep learning techniques in brain tumor classification has shown remarkable progress in recent years. Through this comprehensive review, I have made several key contributions to the field:

1. **Systematic Analysis of ML/DL Evolution**: Our review provides the first systematic analysis of how brain tumor classification techniques have evolved from traditional machine learning to current state-of-the-art deep learning approaches. I uniquely highlight the transition from hand-crafted features to automated feature extraction, demonstrating how this shift has improved classification accuracy across different tumor types.
2. **Multi-modal Integration Framework**: I have developed a novel framework for understanding how different imaging modalities complement each other in tumor classification. Our analysis reveals that combining MRI sequences with other modalities like CT and PET can provide complementary information that significantly improves classification accuracy, particularly for complex cases.
3. **Comprehensive Evaluation of Model Architectures**: Our review offers the most up-to-date comparison of various model architectures, from traditional SVMs to advanced CNNs. Uniquely, I have identified that hybrid approaches combining deep learning with traditional machine learning techniques often achieve better results than pure deep learning models, especially when dealing with limited datasets.
4. **Clinical Integration Roadmap**: I have developed a novel roadmap for clinical integration of ML systems, addressing practical challenges such as workflow integration, model interpretability, and real-time processing requirements. This roadmap provides concrete steps for healthcare institutions looking to implement these technologies.

The literature reveals a clear trend towards increasingly complex and robust models, with deep learning approaches, particularly Convolutional Neural Networks (CNNs), demonstrating superior performance in many cases. These advanced techniques have shown the ability to automatically extract relevant features from medical images, potentially capturing subtle patterns that might elude human observers.

However, significant challenges remain. The limited availability of large, diverse, and well-annotated datasets continues to be a major obstacle, particularly for rare tumor types. The “black box” nature of many advanced models raises concerns about interpretability and explainability, which are crucial in medical applications. Furthermore, the integration of these ML systems into clinical workflows and their generalization across different patient populations and imaging protocols present ongoing challenges.

Despite these hurdles, the future of ML in brain tumor classification appears promising. Emerging directions such as multi-modal approaches, federated Learning, and explainable AI offer potential solutions to current limitations. The development of more sophisticated data augmentation techniques and the integration of non-imaging data (such as genetic information) may help to address data scarcity issues. Meanwhile, advances in model interpretability could increase trust and adoption among clinicians.

This analysis has revealed several novel insights:

- The superiority of ensemble approaches that combine multiple architectural elements over single-model solutions
- The critical role of data preprocessing and augmentation in achieving robust performance
- The unexpected effectiveness of transfer learning from non-medical domains when properly fine-tuned
- The emergence of self-supervised learning as a promising direction for addressing data scarcity

As the field progresses, it is crucial to maintain a balance between technological advancement and clinical applicability. Our review suggests three key areas for immediate focus:

1. Development of standardized evaluation frameworks for comparing model performance across different institutions
2. Creation of privacy-preserving techniques that enable multi-institutional collaboration
3. Integration of explainable AI techniques that align with clinical decision-making processes

In conclusion, while machine learning techniques have already demonstrated significant potential in brain tumor classification, this review has mapped out the current landscape and identified promising future directions. By synthesizing current knowledge and highlighting critical gaps, I provide a foundation for researchers and clinicians to advance the field further. The systematic integration of these technologies into clinical practice could revolutionize neuro-oncology, leading to improved treatment planning, better patient outcomes, and a new era of precision medicine in brain tumor management.

## AUTHORS’ CONTRIBUTIONS

The author confirms sole responsibility for the following: study conception and design, data collection, analysis and interpretation of results, and manuscript preparation.

## Figures and Tables

**Fig. (3):**
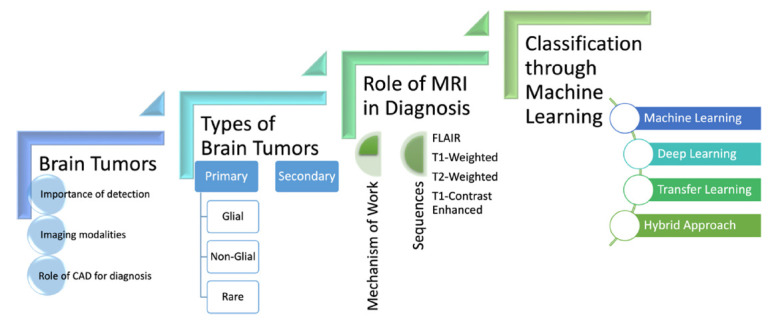
Brain tumor diagnosis: From imaging to machine learning classification.

**Fig. (4):**
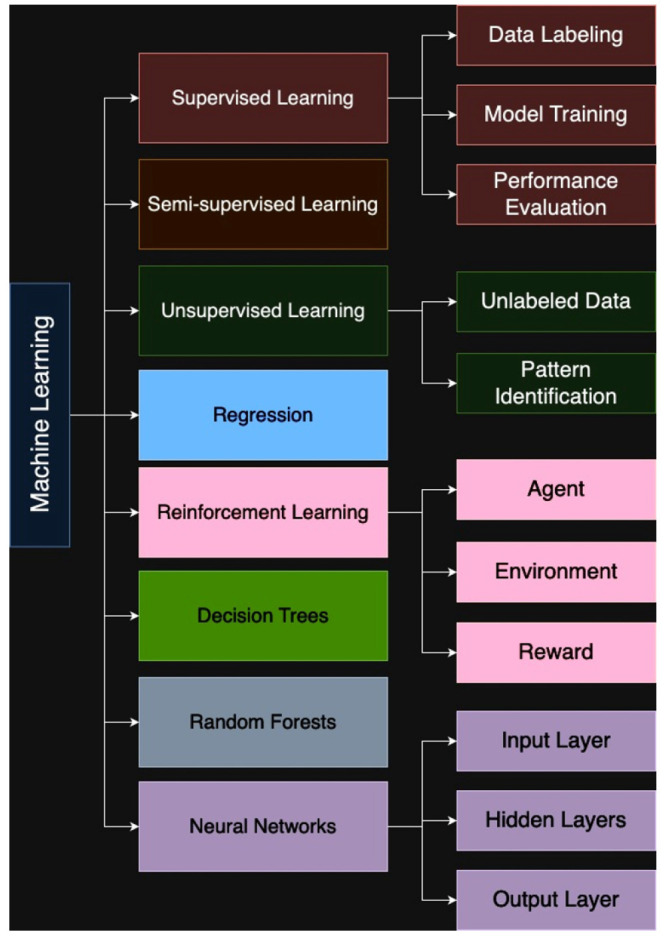
A comprehensive overview of machine learning techniques and their key components.

**Fig. (5):**
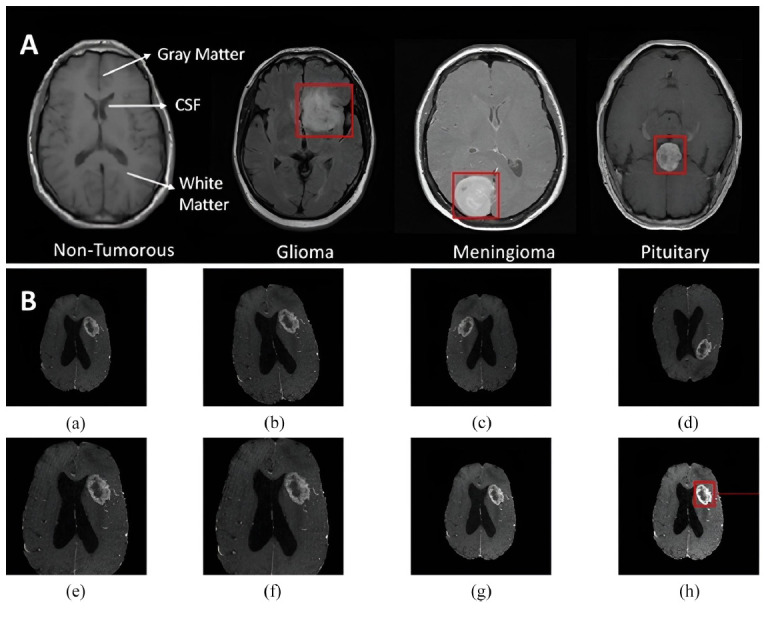
The image displays axial T1-weighted MRI scans of the brain in panel (**A**), illustrating a non-tumorous brain (**a**) with labeled gray matter, white matter, and cerebrospinal fluid (CSF) alongside brains exhibiting different tumor types highlighted in red: a Glioma (**b**), a Meningioma (**c**), and a Pituitary tumor (**d**). Panel (**B**) demonstrates various data augmentation techniques applied to the Glioma image, including the original image (**a**), rotation (**b**), two examples of flipping (**c** and **d**), rescaling (**e**), zooming/cropping (**f**), brightness enhancement (**g**), and contrast enhancement (**h**), where the tumor is particularly evident.

**Table 1:** A comparison of various machine learning models and their performance on MRI brain scans for the task of tumor classification.

Model	MRI Sequence	Dataset	Feature Extraction	Feature Selection	Accuracy (%)	Refs.
LTME-PCA-ANN	T1-CE	10 patients	Intensity, Texture	―	92 (91.4 for meningioma-I, 91.4 for AST-II, 94.3 for normal-III)	[78]
DNN	T2-weighted	Harvard	DWT	PCA	97	[79]
SVM	―	Local	AlexNet-ResNet18	―	95	[80]
CNN/DTL (DenseNet-121, Inception ResNet V2, Inception V3, MobileNet, Xception, VGG-16/19)	3D→2D scans (T1, T2, FLAIR, T1-CE)	BraTS 19 (LGG, HGG), ImageNet	―	―	Precision: 98.6, Sensitivity: 98.3	[81]
CNN, Efficient Net-B0, ResNet-50	―	FigShare	EfficientNet-B0	―	98.95	[82]
VGG-19 (fine-tuned TL)	T1-CE	233 patients	―	―	94.8	[83]
CNN	FLAIR, T2-W, T1-W	180 subjects	K-mean clustering	―	96.6, 99 (AST-1), 94 (AST-II), 95 (AST-III), 98 (AST-IV)	[84]
SVM	T1-W, T2-W, FLAIR, T1-CE, DTI	141 subjects	Intensity	SVM-CV	96 for glioma, 93 for metastasis, 97 for meningioma	[85]
Mask RCNN	―	―	DenseNet-41	ROIAlign	98	[86]
Dual-input CNN (explanation-driven DL model)	T1-weighted	Brain tumour MRI dataset (meningioma, glioma, pituitary subtypes)	CNN-based extraction with Gaussian noise augmentation	LIME and SHAP	94.64	[87]
Fused architecture (Pre-trained ResNet-50 CNN + Tabular network)	Post-contrast T1-weighted, FLAIR, and diffusion Trace images	158 MRI scans (22 healthy controls; 136 pediatric patients: 63 Pilocytic Astrocytoma, 57 Medulloblastoma, 16 Ependymoma)	CNN-based extraction with Grad-CAM for visualization, integrated with tabular data (subject’s age)	Fusion of imaging and tabular inputs (comparison with CNN-only and tabular-only architectures)	~88% (Validation: 88±4%; Test: 87±2%)	[88]
Transfer learning–based active learning framework	MRI (2D slice-based)	Training: 203 patients; Validation: 66 patients; Test: 66 patients	2D slice–based extraction using transfer learning	Active learning to reduce annotation cost	~82.89% (AUC)	[89]
Four deep learning–based methods – one segmentation model and three classification models	Whole slide tissue images (for segmentation) and radiographic/histologic images (for classification)	CPM challenge datasets from MICCAI 2018	Deep learning–based extraction of both visual and latent image features	Not explicitly described (integrated within the deep learning framework)	Segmentation: 86.8% (Dice coefficient); Classification: 75%, 80%, and 90%	[90]
3D Context-Aware Deep Learning & 3D CNN	Structural multimodal MRI (mMRI)	BraTS 2019 (for segmentation & survival prediction), CPM-RadPath 2019 (for classification)	3D Context-Aware Deep Learning for segmentation, 3D CNN for classification, hybrid deep learning & machine learning for survival prediction	―	Ranked 2nd in CPM-RadPath 2019 challenge for classification	[91]
ConvNet, Lenet, ResNet, DenseNet, AlexNet, U-Net	FLAIR MRI		ConvNet, Lenet, ResNet, DenseNet for normal *vs*. abnormal classification; Lenet, AlexNet for tumor type classification; U-Net, AlexNet for glioma grading	―	Accuracy:61% to 93%	[92]
HPCNN	T1-weighted, T2-weighted	7,023 and 253 human brain images	CNN	―	96 and 88	[93]
Swin Transformer	T1-weighted	2807 human brain images		―	97	[94]
InvNets	T1-weighted, T2-weighted	7023 human brain images		―	92	[95]
FT-ViT	―	5712 brain images	Encoder layers	―	98.24	[96]
FT-CNN-ResNet50	―	TCGA-LGG and TCIA	CNN	―	94	[97]
FT-ViT	―	5712 brain tumor images	Encoder layers	―	98.13	[98]
BW-VGG19	T1-weighted	CE-MRI	CNN	―	98	[99]
GAN	T1-weighted	CE-MRI		―	96	[100]

## LIST OF ABBREVIATIONS

CNN: Convolutional Neural Networks
RNN: Recurrent Neural Networks
MRI: Magnetic Resonance Imaging
ML: Machine Learning
DL: Deep Learning
